# Impact of Obstructive Sleep Apnea-Hypopnea Syndrome Severity on Heart Rate Variability and QTc Interval in Hypertensive Patients

**DOI:** 10.3390/medicina61122221

**Published:** 2025-12-16

**Authors:** Milovan M. Stojanović, Marina Deljanin Ilić, Lidija Ristić, Zoran Stamenković, Goran Koraćević, Dejana Gojković, Jovana Kostić

**Affiliations:** 1 Department for Cardiovascular Diseases, Institute for Treatment and Rehabilitation Niška Banja, 18000 Nis, Serbia; marinadi@mts.rs (M.D.I.); dejana.gojkovic1997@gmail.com (D.G.); j.kostic1998@gmail.com (J.K.); 2 Faculty of Medicine, Niš University, 18000 Nis, Serbia; ristic60lidija@gmail.com (L.R.); gkoracevic@yahoo.com (G.K.); 3 Clinic for Lung Diseases, University Clinical Center Niš, 18000 Nis, Serbia; zokinis@live.com; 4 Clinic for Cardiovascular Diseases, University Clinical Center Niš, 18000 Nis, Serbia

**Keywords:** obstructive sleep apnea–hypopnea syndrome, arterial hypertension, heart rate variability, QTc interval, sudden cardiac death, apnea–hypopnea index

## Abstract

*Background and Objectives*: Obstructive Sleep Apnea–Hypopnea Syndrome (OSAHS) is associated with increased cardiovascular risk, particularly in hypertensive patients. Heart rate variability (HRV) and QTc interval are noninvasive markers of autonomic function and ventricular repolarization, respectively, but their relationship with OSAHS severity remains unclear. To investigate whether the severity of OSAHS influences standard HRV and QTc parameters in hypertensive patients with moderate or severe OSAHS. *Materials and Methods*: This prospective study included 110 hypertensive patients with moderate (AHI 15–29.9/h, *n* = 37) or severe (AHI ≥ 30/h, *n* = 73) OSAHS. All patients underwent full-night respiratory polygraphy and 24-h Holter ECG monitoring. HRV indices (SDNN, SDANN, SDNNi, RMSSD, SDSD) and QTc interval were analyzed. Associations with OSAHS parameters and nocturnal hypoxemia were assessed using partial correlation and multivariate models, adjusted for obesity, diabetes mellitus, metabolic syndrome, and beta-blocker use. *Results*: Patients with severe OSAHS had higher body weight and neck circumference, and a higher prevalence of diabetes and obesity compared to those with moderate OSAHS. HRV and QTc parameters did not differ significantly between groups. Notably, reduced SDNN was independently associated with the percentage of time spent with oxygen saturation below 90% (TST90%, *p* = 0.003) and mean oxygen saturation (*p* = 0.003), indicating autonomic imbalance. QTc prolongation (≥450 ms in men, ≥460 ms in women) was present in 9.6% of patients but was not directly related to OSAHS severity. *Conclusions*: Hypertensive patients with OSAHS have a high cardiovascular risk burden and frequent autonomic dysfunction. Nocturnal hypoxemia is independently associated with impaired HRV, reflecting sympathetic predominance. QTc prolongation appears to be influenced by additional comorbidities rather than OSAHS severity alone.

## 1. Introduction

Obstructive Sleep Apnea–Hypopnea Syndrome (OSAHS) is the most common sleep-related breathing disorder (SRBD) [1]. It is characterized by recurrent episodes of partial or complete collapse of the upper airway during sleep despite preserved respiratory effort [1]. If not diagnosed and adequately treated, OSAHS may lead to insulin resistance, obesity, metabolic syndrome, diabetes mellitus, and nonalcoholic fatty liver disease [2]. Beyond these metabolic consequences, OSAHS also (directly and indirectly) increases the risk of cardiovascular diseases (CVDs) [3] and is recognized as an independent risk factor for arterial hypertension (HTA), atrial fibrillation (AF), heart failure (HF), coronary artery disease (CAD), and stroke [4,5,6,7]. The pathophysiological mechanisms initiated and potentiated by OSAHS that contribute to CVD development are numerous and complex.

Patients with severe OSAHS have an increased risk of both all-cause and cardiovascular mortality [8], whereas this association is not evident in those with mild or moderate diseases. In the United States alone, approximately 38,000 deaths annually are attributed to OSAHS [9]. Moreover, the Wisconsin Sleep Cohort Study revealed that individuals with severe OSAHS have a very high risk for cardiovascular (hazard ratio of 5.2) and all-cause mortality (hazard ratio of 3.8) [10]. The excess mortality observed in OSAHS may be explained by the increased prevalence of metabolic and cardiovascular comorbidities within this population [11]. Furthermore, OSAHS has been identified as an independent risk factor for sudden cardiac death (SCD) [12], attributed to a complex interplay of mechanisms including myocardial ischemia, autonomic nervous system dysfunction, altered ion channel expression, and disturbances in ventricular repolarization [13,14].

A prolonged QTc interval is a well-established risk factor for SCD [15], primarily by promoting the development of malignant arrhythmias due to altered ventricular repolarization. The risk of such dysrhythmic events rises progressively with increasing QTc duration [16]. Importantly, greater OSAHS severity has been shown to correlate with the degree of QT interval prolongation [17]. These findings suggest that the elevated prevalence of SCD in patients with OSAHS may, at least in part, be mediated by QTc prolongation.

Several studies have demonstrated that OSAHS is also associated with alterations in autonomic nervous system (ANS) function, primarily driven by intermittent hypoxia [18]. This ANS dysfunction is typically characterized by sympathetic overactivity. Moreover, the observed link between OSAHS severity and sympathetic dysregulation indicates a dose–response relationship, with more severe forms associated with greater sympathetic activation [18]. Heart rate variability (HRV) is a well-recognized, noninvasive marker of ANS imbalance and provides valuable insight into the cardiac autonomic control mechanisms [19].

Despite the strong evidence linking OSAHS to cardiovascular diseases, the impact of OSAHS severity on both ventricular repolarization and autonomic dysfunction in hypertensive patients remains insufficiently explored. Most available studies included heterogeneous populations (with and without HTA, CAD, HF, etc.) and typically focus only on a single parameter—HRV or QTc [20,21]. Comorbidities in OSAHS may alter HRV and QTc indices, and this is why the extent to which OSAHS severity itself influences these parameters remains unclear. This gap enables us to understand whether OSAHS severity exerts an independent impact on ventricular repolarization and autonomic dysfunction in hypertensive patients.

The aim of this study was to investigate whether the severity of OSAHS influences standard HRV and QTc parameters in hypertensive patients with moderate or severe OSAHS.

## 2. Materials and Methods

### 2.1. Study Design and Patient Selection

**Figure 1:**
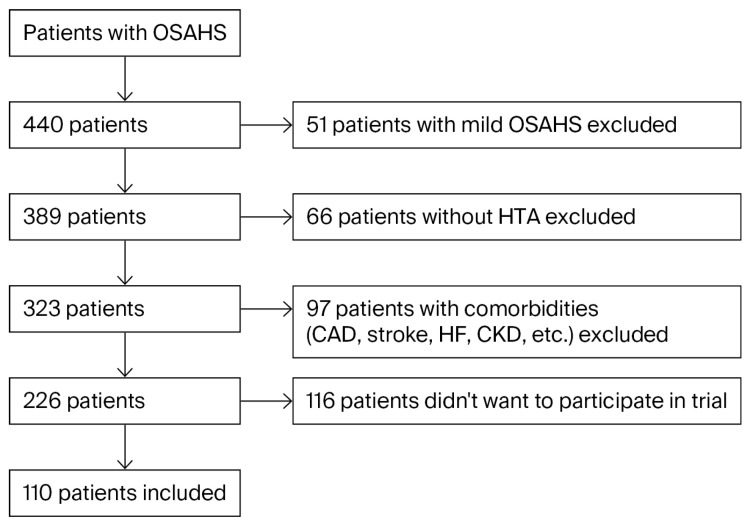
PRISMA-like flow chart: exclusion criteria during study randomization.

Out of 440 consecutive OSAHS patients, 110 hypertensive patients with moderate (AHI between 15 and 29.99 per hour, 37 patients (33.6%)) or severe (AHI ≥ 30 per hour, 73 patients (66.4%)) OSAHS were included in this prospective study due to strict exclusion criteria (Figure 1). The diagnosis of OSAHS was made based on the full-night respiratory polygraphy (RPG) performed by the ALICE NightOne, Philips Respironics (Murrysville, PA, USA) device at the sleep laboratory at the Clinic for Lung Diseases University Clinical Center, Nis, during the patient’s habitual sleep time and by using criteria from an American Academy of Sleep Medicine (AASM) Clinical Practice Guideline for Diagnostic Testing for Adult Obstructive Sleep Apnea [22]. The RPG parameters used in the study were AHI, oxygen desaturation index (ODI), time spent with oxygen saturation below 90% (TST90%), and minimal, average, and maximal oxygen saturation, graded according to AASM recommendations and criteria.

Patients with known CAD, HF, severe valvular disease, or an artificial valve, chronic kidney disease (defined as an estimated glomerular rate below 60 mL/min/1.73 m^2^), patients younger than 40 years or older than 80 years, and patients with impaired physical or mental condition were not included in the study.

### 2.2. Parameters of Holter ECG

After finishing the sleep study, all patients were hospitalized at the Department for Cardiovascular Diseases at the Institute for Treatment and Rehabilitation, Niska Banja, where clinical assessment and anthropometric measurements were performed. Data on cardiovascular risk factors, including hypertension, dyslipidemia, stress, smoking, diabetes mellitus, obesity, physical inactivity, and family history of cardiovascular disease, were also collected.

All patients underwent 24-h Holter electrocardiographic (ECG) monitoring during hospitalization at the Institute, using a standard three-channel device (DEL MAR, TN, USA). All recordings were obtained within one month of establishing the diagnosis of OSAHS, and in treatment-naïve patients. The analysis included rhythm assessment (sinus rhythm or atrial fibrillation), minimum, mean, and maximum heart rate (HR) values, episodes of bradycardia (defined as average HR < 60 bpm or night HR < 40 bpm) or pauses (defined as ≥2 s), and the occurrence of arrhythmias or conduction disturbances.

Heart rate variability (HRV) analysis was performed using the following parameters: standard deviation of all normal-to-normal intervals (SDNN, normal > 100 ms), root mean square of successive differences (RMSSD), standard deviation of successive differences (SDSD), mean of the standard deviations of all NN intervals in 5-min segments (SDNNi), and standard deviation of the averages of NN intervals across all 5-min segments (SDANN, normal > 100 ms). SDNN is the most widely used marker of overall HRV and reflects both sympathetic and parasympathetic influences. Lower values of SDNN (<100 ms) have been associated with a higher risk for adverse cardiovascular events. On the other hand, SDANN usually reflects long-term (circadian) components of HRV, while SDANNi represents a short-time variability and is more sensitive marker of parasympathetic activity. SDSD and RMSSD are short-term (beat-to-beat) variability markers and are strongly influenced by parasympathetic activity.

QT interval analysis included measurement of the QT interval, corrected QT interval (QTc; normal ≤ 450 ms in men and ≤ 460 ms in women), RR interval, and T-wave amplitude (TAMP). The QTc interval was calculated using Bazett’s formula (QTc = QT/√RR). In patients with atrial fibrillation (*n* = 8), HRV analysis was not performed, whereas QTc analysis was conducted. Conversely, in patients with bundle branch block (*n* = 6), QTc analysis was not performed, but HRV analysis was carried out. All Holter ECG parameters were analyzed according to the latest Heart Rhythm Society guidelines [23].

### 2.3. Statistical Analysis

Data are presented as standard descriptive statistics (mean, median, standard deviation, and interquartile range) or as frequencies and percentages. Comparisons of numerical variables between groups were performed using the *t*-test or the Mann–Whitney test. Comparisons of categorical data were performed by using the Chi-squared test. Firstly, the association between sleep apnea and cardiovascular parameters was estimated using partial correlation analyses, controlling for specific clinical parameters. Secondly, the significant parameters from the partial correlation analysis were included in a multivariate linear regression model. A null hypothesis was tested at a *p*-value < 0.05. Statistical analysis was performed by R 4.5.1 (Foundation for Statistical Computing, Vienna, Austria) and Posit RStudio 2025.09.1+401 (PBC, Boston, MA, USA) [24,25].

The sample size was calculated under the assumption of a moderate effect size (r = 0.3) between SDNN and sleep apnea parameters, while controlling for four variables in a partial correlation analysis. With a study power of 95%, an alpha error of 0.05, and a two-tailed test of the null hypothesis, the required sample size was 89 patients. Sample size calculation was performed in G*Power 3.1.9.7 (linear multiple regression, fixed model R^2^ increases). Due to possible drop-outs during the study, the calculated sample size was increased by 20% to 107 patients, and then rounded up to 110 to ensure adequate power of the study.

## 3. Results

The groups were comparable in terms of age and sex (*p* = 0.763 and *p* = 0.381, respectively). An anthropometric characteristic that differed significantly between the examined groups was neck circumference (*p* = 0.008) (Table 1).

Comparison of the prevalence of cardiovascular risk factors revealed that obesity (body mass index ≥ 30 kg/m^2^) was significantly more common among patients with severe OSAHS (*p* = 0.033) (Table 2).

Comparison of parameters obtained from 24-h Holter ECG monitoring revealed no statistically significant differences between the examined groups. A high prevalence of cardiac pauses was observed in both groups (8.1% vs. 6.8%). Likewise, both groups demonstrated a low prevalence of preserved SDNNi (58.8% vs. 58.7%) and SDANN values (62.2% vs. 72.7%) (Table 3).

In the partial correlation analysis, a statistically significant association was found between SDNN and the following sleep apnea parameters: percentage of time spent with oxygen saturation below 90% (r = −0.282, *p* = 0.007) and mean oxygen saturation (r = 0.321, *p* = 0.002) (Table 4). On the other hand, no statistically significant association between QTc values and sleep apnea parameters was found after adjustment for obesity, diabetes mellitus, metabolic syndrome, and beta-blocker use (Table 4).

Using a multivariate model, a statistically significant negative association was found between SDNN and the percentage of time spent with oxygen saturation below 90% (*p* = 0.003) and beta-blocker use (*p* = 0.015) in Model 1 (Table 5). Additionally, in multivariate Model 2, there was a statistically significant positive association between SDNN and mean oxygen saturation (*p* = 0.003) and a negative association between SDNN and beta-blocker use (*p* = 0.007).

## 4. Discussion

In the present study, patients with severe OSAHS had higher neck circumference compared to those with moderate disease. Moreover, obesity was more prevalent among patients with severe OSAHS. Both groups demonstrated a high overall prevalence of cardiovascular risk factors. No statistically significant differences were observed between the examined groups regarding HRV and QT interval parameters. Nevertheless, a high prevalence of SDANN and SDNNi impairment was noted in both groups, indicating autonomic imbalance. Importantly, we found significant associations between SDNN and the percentage of time spent with oxygen saturation below 90% (TST90%) (*p* = 0.003), as well as between SDNN and mean oxygen saturation (*p* = 0.003). In contrast, no significant correlations were found between QTc and any of the sleep apnea indices.

HRV is a widely used, noninvasive method for evaluating ANS function. By analyzing oscillations in consecutive R–R intervals, HRV provides insight into the dynamic balance between sympathetic and parasympathetic influences on sinoatrial node activity [26]. Higher HRV values are generally favorable and have been associated with better overall health and reduced risk of SCD [27]. On the other hand, reduced HRV reflects autonomic dysfunction and has been linked to impaired physical fitness and a greater risk of cardiovascular and neurodegenerative disorders [28]. In this context, increased HRV typically signifies effective recovery and adaptation, whereas decreased HRV may indicate physiological stress or underlying disease processes [29].

Sleep-related breathing disorders have been consistently associated with impaired HRV, even among young and otherwise healthy individuals [30]. This adverse effect is particularly pronounced in patients with OSAHS [31]. Intermittent hypoxia and hypercapnia are considered major contributors to ANS dysfunction in OSAHS [32]. These factors, combined with recurrent arousals and negative intrathoracic pressure swings, promote chronic sympathetic overactivation and, thereafter, autonomic dysregulation. Consequently, patients with OSAHS frequently exhibit lower SDNN and SDANN values compared with healthy controls [31,33].

In OSAHS, apneic events are usually accompanied by bradycardia followed by an abrupt rise in heart rate upon apnea termination. This characteristic heart rate oscillation is known as cyclic variation in heart rate (CVHR) [34]. The CVHR frequency positively correlates with AHI [35,36], and some authors have proposed using CVHR as a screening tool in identifying patients with moderate to severe OSAHS [37]. On the other hand, CVHR amplitude is primarily mediated by the vagus nerve and is reduced in patients with autonomic dysfunction [34]. It is of utmost importance as decreased CVHR amplitude is an independent prognosticator of cardiovascular mortality [38]. This implies that both CVHR amplitude and frequency are essential in assessing autonomic dysfunction in OSAHS patients. On the other hand, standard HRV parameters commonly used in everyday clinical practice are reliable markers of autonomic dysfunction [39], especially in patients with sleep-related breathing disorders [40].

Time-domain HRV indices characterize overall ANS balance (SDNN, SDANN) and short-term autonomic modulation (SDNNi, RMSSD, SDSD), allowing assessment of autonomic (dys)function. Our results support the concept of autonomic imbalance in OSAHS, demonstrated by the high prevalence of SDANN and SDNNi impairment in both study groups. The altered HRV indices in patients with moderate OSAHS support the concept that ANS dysfunction develops early in the course of OSAHS. The ANS impairment can precede structural cardiac changes and may explain why even patients with moderate OSAHS have an increased cardiovascular risk. Additionally, the observed associations between reduced SDANN values and hypoxemia parameters (TST90% and mean oxygen saturation) highlight the crucial role of nocturnal hypoxia in autonomic dysfunction. Intermittent hypoxia leads to a cascade of physiological responses that may amplify sympathetic discharge even during wakefulness. The increased sympathetic tone is usually accompanied by reduced vagal activity which results in diminished parasympathetic-mediated HRV indices. If this repeats every night, it can lead to permanent autonomic dysfunction and sympathetic predominance which can influence long-term indices such as SDNN and SDANN. Also, these findings reinforce the notion that assessing OSAHS severity solely based on AHI may be insufficient and somewhat outdated [41]. Incorporating markers of oxygen desaturation and autonomic activity could provide a more comprehensive evaluation of cardiovascular risk in these patients. In addition, the observed correlation between nocturnal hypoxemia and HRV impairment suggests that therapeutic strategies that effectively reduce oxygen desaturation, like weight loss, CPAP, or positional therapy, may improve autonomic function in OSAHS patients.

Several trials have examined ANS dysfunction in patients with OSAHS, but most included heterogeneous population with (multiple) comorbidities. Our results, derived from relatively homogenous cohort of patients without structural heart disease, emphasize that ANS dysfunction is present even in OSAHS patients without CVD. Furthermore, our findings suggest that the impairment of ANS may represent one of the earliest consequences of OSAHS [42], and could serve as a mechanistic link between OSAHS and CVDs such as arrhythmias or conduction disorders.

QT prolongation in OSAHS is multifactorial. Intermittent hypoxia, large intrathoracic pressure swings, and sympathetic overactivation may lead to altered ventricular repolarization. Furthermore, oxidative stress and inflammation may further contribute to delayed repolarization, thereby increasing the risk of arrhythmias and SCD. In this study, 10 out of 104 patients (9.62%) had prolonged QTc values (≥450 ms for males, ≥460 ms for females) on Holter ECG. This high prevalence of prolonged QTc interval is even more noteworthy considering that this study included only patients without comorbidities commonly associated with QT prolongation, such as structural heart diseases, CAD, or renal failure. Furthermore, all included patients were receiving only antihypertensive therapy, among which only beta-blockers have a meaningful effect on QTc. The analyses related to beta-blocker use were performed and are presented in our study. The high prevalence of prolonged QTc highlights the possible cause for prevalent cardiac arrhythmia and sudden cardiac death in patients with OSAHS [43].

In contrast to HRV indices, our study did not demonstrate a significant association between QTc interval and the severity of OSAHS or any of the sleep apnea parameters after adjustment for major confounders. Previous studies have reported conflicting results regarding the relationship between OSAHS severity and QTc interval. Some authors found a positive correlation between apnea burden and QTc prolongation, particularly in severe disease and in the presence of comorbid cardiovascular conditions [12,13,17]. Others, however, failed to confirm this association when controlling for confounding factors such as obesity, diabetes mellitus, and medication use [44,45]. Therefore, our findings are consistent with the latter group of studies, suggesting that the impact of OSAHS on ventricular repolarization is likely modulated by coexisting clinical and pharmacological variables. The complexity of assessing the repolarization abnormalities in OSAHS patients highlights the need for longitudinal studies incorporating electrophysiological markers.

### 4.1. Study Limitations

Our Holter ECG device does not support automated detection of CVHR. Therefore, we were unable to interpret both CVHR amplitude and frequency, which are very important in assessing autonomic dysfunction in OSAHS. Our conclusions are based on standard HRV parameters. Future studies incorporating CVHR analysis will be important for delineating apnea–hypopnea-related autonomic disturbances more precisely.

### 4.2. Strengths of This Study

This study included only hypertensive OSAHS patients without comorbidities commonly associated with QT prolongation or impaired HRV, such as structural heart disease, coronary artery disease, or renal dysfunction. In addition, all participants were receiving exclusively antihypertensive therapy, among which only beta-blockers have a meaningful impact on QTc duration and HRV indices. Analyses related to beta-blocker use were performed and are presented in our results.

## 5. Conclusions

Patients with OSAHS and hypertension exhibit a high cardiovascular risk burden, accompanied by a considerable prevalence of impaired HRV indices and prolonged QTc intervals. Nocturnal hypoxemia was independently associated with reduced HRV, reflecting autonomic imbalance. In contrast, QTc prolongation was not directly related to OSAHS severity, suggesting that ventricular repolarization abnormalities may be influenced by additional comorbid and clinical factors.

## Figures and Tables

**Table 1:** Anthropometric parameters.

	AHI 15–29.9		AHI ≥ 30		*p* ^1^
Age (years)	54.05 ± 8.76		53.49 ± 9.39		0.763
Gender *n* (%)					
Male	27	73.0	60	82.2	0.381 ^2^
Female	10	27.0	13	17.8	
Weight (kg)	106.61 ± 19.68		113.86 ± 16.70		0.063
Height (cm)	175.72 ± 11.01		176.67 ± 7.83		0.648
Waist circumference (cm)	119.44 ± 14.64		122.96 ± 12.15		0.190
Neck circumference (cm)	44.14 ± 4.07		46.25 ± 3.63		0.008
Body mass index (kg/m^2^)	34.54 ± 5.95		36.68 ± 5.17		0.069

Mean ± SD; ^1^ Mann–Whitney test; ^2^ *t*-test.

**Table 2:** Risk factors for cardiovascular diseases.

	AHI 15–29.9		AHI ≥ 30		*p* ^1^
	N	%	N	%	
Smoking	21	56.8	37	50.7	0.689
Physical inactivity	13	35.1	34	46.6	0.346
Obesity	29	78.4	67	93.1	0.033 ^2^
Stress	12	32.4	15	20.5	0.257
Diabetes mellitus	6	16.2	25	34.2	0.072
Heredity	24	64.9	41	56.2	0.502
Dyslipidemia	28	75.7	48	65.8	0.398
Number of risk factors (Median, IQR)	4	3–8	5	4–6	0.697 ^2^

^1^ Chi-square test; ^2^ Mann–Whitney test; IQR—interquartile range.

**Table 3:** Parameters of 24 h Holter ECG.

	AHI 15–29.9		AHI ≥ 30		*p* ^1^
Parameters	*n*	(%)	*n*	(%)	
Minimal HR ^†^	54.76 ± 8.47		53.97 ± 7.67		0.628
Maximal HR ^†^	112.57 ± 15.72		113.25 ± 15.98		0.833
Average HR ^†^	72.35 ± 10.67		71.63 ± 10.32		0.733
Pauses	3	8.1	5	6.8	1.000 ^2^
Bradycardia	6	16.2	18	24.7	0.442 ^2^
SDNN ^†^	129.52 ± 36.12		130.2 ± 33.94		0.674 ^1^
SDNN > 100 ms	31	83.8	54	83.1	1.000 ^2^
RMSD ^†^	50.31 ± 31.3		44.43 ± 18.64		0.736 ^1^
SDSD ^†^	42.01 ± 27.92		36.32 ± 16.06		0.689 ^1^
SDNNi ^†^	44.62 ± 15.83		43.63 ± 14.05		0.571 ^1^
SDNNi > 40 ms	20	58.8	37	58.7	1.000 ^2^
SDANN ^†^	113.3 ± 35.41		121.57 ± 32.21		0.067 ^1^
SDANN > 100 ms	23	62.2	48	72.7	0.374 ^2^
RR ^†^	857.66 ± 128.6		868.81 ± 115.69		0.567 ^1^
QT ^†^	417.09 ± 92.83		397.4 ± 24.7		0.339 ^3^
QTc ^†^	413.76 ± 34.6		409.46 ± 29.68		0.688 ^3^
TAMP ^†^	0.17 ± 0.07		0.17 ± 0.08		0.569 ^3^

^†^ Arithmetic mean ± SD; ^1^ Mann–Whitney test; ^2^ Chi-square test; ^3^ *t*-test. Legend: HR—heart rate; SDNN—standard deviation of all normal-to-normal intervals; RMSSD—root mean square of successive differences; SDSD—standard deviation of successive differences; SDNNi—mean of the standard deviations of all NN intervals in 5-min segments; SDANN—standard deviation of the averages of NN intervals across all 5-min segments; TAMP—T-wave amplitude.

**Table 4:** Association of SDNN and QTc with sleep apnea parameters (partial correlation analysis).

Parameters		SDNN	QTc
Apnea–hypopnea index	r	−0.057	−0.076
	*p*	0.594	0.475
	df	88	88
Oxygen desaturation index per hour	r	−0.112	0.043
	*p*	0.295	0.689
	df	88	88
Time spent with oxygen saturation below 90%	r	−0.282	−0.159
	*p*	0.007	0.135
	df	88	88
Minimal oxygen saturation	r	0.006	−0.098
	*p*	0.953	0.356
	df	88	88
Average oxygen saturation	r	0.321	0.144
	*p*	0.002	0.176
	df	88	88
Control variables: obesity, diabetes mellitus, metabolic syndrome, beta blocker			

r—correlation coefficient; df—degree of freedom.

**Table 5:** Association between SDNN and percentage of time spent with oxygen saturation below 90% in the multivariate model—multivariate linear regression analysis.

Models	B	SE	Beta	*p*
Model 1				
(Constant)	139.439	13.053		0.000
Obesity	0.316	11.508	0.003	0.978
Diabetes mellitus	8.644	7.757	0.111	0.268
Metabolic syndrome	17.481	8.966	0.195	0.054
Beta blocker use	−19.495	7.875	−0.240	0.015
TST 90%	−0.376	0.122	−0.309	0.003
Dependent variable: SDNN. Adjusted R^2^ = 0.144				
Model 2				
(Constant)	−118.917	84.751		0.164
Obesity	6.070	10.162	0.058	0.552
Diabetes mellitus	7.068	7.550	0.091	0.352
Metabolic syndrome	13.439	8.488	0.157	0.117
Beta blocker use	−21.245	7.721	−0.261	0.007
Average oxygen saturation	2.732	0.887	0.300	0.003
Dependent variable: SDNN. Adjusted R^2^ = 0.148				

B—regression coefficient; SE—standard error; Beta—standardized regression coefficient. Legend: TST 90%—time spent with oxygen saturation below 90%.

## Data Availability

Research data are not publicly available due to privacy restrictions.
